# A preliminary survey reveals that common viruses are found at low titers in a wild population of honey bees *(Apis mellifera)*

**DOI:** 10.1093/jisesa/iead117

**Published:** 2023-12-14

**Authors:** Myra Dickey, Mckaela Whilden, Jordan Twombly Ellis, Juliana Rangel

**Affiliations:** Department of Entomology, Texas A&M University, College Station, TX, USA; Department of Entomology, Texas A&M University, College Station, TX, USA; Department of Entomology, Texas A&M University, College Station, TX, USA; Department of Entomology, Texas A&M University, College Station, TX, USA

**Keywords:** black queen cell virus (BQCV), deformed wing virus (DWV), feral honey bees, honey bee-associated viruses, unmanaged colonies

## Abstract

A major threat to honey bee (*Apis mellifera* Linnaeus, Hymenoptera: Apidae) health continues to be parasitism by the mite *Varroa destructor*, which has been linked to high colony losses worldwide. Besides feeding on developing and adult bees, *Varroa* is also a prolific vector of honey bee-associated viruses. Because they live in unmanaged conditions, wild honey bee colonies are not treated against *Varroa*, which has enabled the natural selection of more mite-tolerant bees. To date, few studies have explored the prevalence of viruses in unmanaged colonies. The Welder Wildlife Refuge (WWR) in Texas is a unique site to study the viral landscape of unmanaged honey bees in the United States. The goals of this study were to identify and quantify viruses in wild colonies at the WWR, to examine changes in the prevalence of viruses in these colonies over time, and to compare the presence and titers of viruses between wild colonies at the WWR and those from the nearest managed apiary. We collected bees from colonies at the WWR in 2013, 2016, and 2021, and analyzed selected viruses for their presence and titers via quantitative polymerase chain reaction. In 2021, we also sampled bees from the nearest managed apiary for comparison. We found low average virus titers in all wild colonies sampled, and no difference in virus titers between colonies at the WWR and those from the managed apiary. Our study indicates that virus titers in wild colonies at the WWR are similar to those found in nearby colonies, and that these titers fluctuate over time.

## Introduction

The western honey bee, Apis mellifera Linnaeus (Hymenoptera: Apidae) is arguably the most widely used beneficial insect for the commercial pollination of major agricultural crops, contributing over $200 billion dollars to the global economy annually (Gallai et al. 2009, Calderone, 2012). But despite their importance, many populations of managed honey bees around the world continue to suffer high rates of overwintering mortality, mostly due to a combination of factors that include parasites, pathogens, pesticide exposure, malnutrition, and poor queen quality (Kulhanek et al. 2017, Bruckner et al. 2022; but see Osterman et al. 2021 on the rise of colony numbers in some locations). Of these factors, the most detrimental threat to honey bee health continues to be the ectoparasitic mite *Varroa destructor*, which has been linked to high colony losses worldwide (Dainat et al. 2012, Mondet et al. 2014, Natsopoulou et al. 2017, Ramsey et al. 2019).

*Varroa* mites are highly effective vectors of several honey bee-associated viruses, which they can transmit while feeding on the fat body tissue of developing and adult honey bees (Ramsey et al. 2019, Beaurepaire et al. 2020). The 2 most common virus complexes that have been linked to *Varroa* transmission are the acute bee paralysis virus (ABPV) complex, which includes Israeli acute paralysis virus (IAPV) and Kashmir bee virus (KBV), as well as the deformed wing virus (DWV) complex, which includes 3 major strains (DWV-A, DWV-B, and DWV-C) (de Miranda et al. 2010, Mordecai et al. 2016, McMahon et al. 2018, Posada-Florez et al. 2019). ABPV and DWV are single-stranded RNA viruses that infect honey bees at all developmental stages (de Miranda et al. 2010). Before the arrival of *Varroa* as a novel parasite of *A. mellifera*, these viruses were present at low titers in most colonies and were considered relatively harmless (Genersch and Aubert 2010, de Miranda et al. 2013, Amiri et al. 2018). However, after the arrival of *Varroa*, colony decline (and ultimately death) has usually been associated with both, high mite infestation levels, and high titers of mite-vectored viruses (Martin et al. 2012, van Dooremalen et al. 2012, Tantillo et al. 2015, Grozinger and Flenniken 2019). While the distinctive signs of DWV infection include wing deformities, which result in flightless adults that die shortly upon emerging (de Miranda and Genersh 2010), those symptoms are almost always seen only through *Varroa-*mediated transmission of DWV in the pupal stages of bee development (Moeckel et al. 2011). There have also been other DWV genotypes identified that have shown reduced symptoms and higher survival rates than the most common strains (Ray et al. 2023). Likewise, high ABPV titers have been linked in some studies with high *Varroa* mite titers in colonies, although it is still unclear if the mite directly transmits this virus complex. Whenever present, ABPV is portrayed by severe pupal mortality, as well as trembling and paralysis in those bees that make it into adulthood (de Miranda et al. 2010).

Not surprisingly, when a colony’s high mite population is not controlled, there is usually a rampant increase in the titers of DWV and other viruses (Martin et al. 2012, van Dooremalen et al. 2012, Tantillo et al. 2015, Grozinger and Flenniken 2019). The titers of honey bee-associated viruses (henceforth referred to as “viruses”) in managed colonies can be detectable in the spring, but typically peak in the late summer to early fall, which correlates with the time of year when *Varroa* infestation is also at its highest (Runckel et al. 2011, Francis et al. 2013, Faurot-Daniels et al. 2020). Despite this reliance of managed beekeeping operations on chemical treatments against *Varroa* to ensure colony survival, the use of miticides has been shown to increase virus quantities in certain settings (Locke et al. 2012).

Interestingly, there are wild populations of honey bees scattered throughout the world that have survived without human intervention, even after the introduction of this pervasive parasite (*Australia*: Chapman et al. 2008; *Europe*: Oleksa et al. 2013; Thompson et al. 2014; *Sweden*: Thaduri et al. 2018, 2019; Locke et al. 2021; *United States*: Schiff et al. 1994; Rangel et al. 2016a; b; Seeley, 2017; Seeley 2019; Rangel et al. 2020). Over the last 30 years, a large population of wild colonies located at the Welder Wildlife Refuge (WWR) near Sinton, TX, has been studied to better understand the biology of unmanaged honey bees in the Southern United States (Pinto et al. 2005, Rangel et al. 2016a, 2016b). A survey of this population from 1991 to 2001 showed that the mitochondrial haplotype frequency of these colonies changed drastically from being solely of Western European maternal lineage in the late 1990s to being dominated by Africanized maternal lineages in the late 2000s (Pinto et al. 2005). A follow-up study showed that the same population is now an admixed swarm with a continued greater presence of the *Apis mellifera scutellata* lineage and low titers of European genetic ancestry (Rangel et al. 2016a).

The success of Africanized honey bees (AHBs) in the Americas has been attributed to a combination of genetic and ecological factors that have conferred them higher fitness compared to some resident populations of European descent (Rubink et al. 1996, Pinto et al. 2004, Schneider et al. 2004). Some of these characteristics include a shorter development period, higher swarming rate, higher absconding rate, lower selectivity when choosing nest sites, lower colony density, smaller nests, and higher defensiveness against predators, pests, and parasites (Winston et al. 1983, Schmidt and Hurley 1995, Caron 2001, Schneider et al. 2004, Utaipanon et al. 2019). These traits, combined with the advantages of living in unmanaged conditions for decades, make AHBs an ideal system for studying the ecology of wild honey bees in the southern United States. Despite the previous findings that the WWR comprises a genetic predominance of AHBs, not much else is known about the presence of pathogens in this unmanaged honey bee population.

The aim of this study was to identify and quantify selected RNA viruses in unmanaged honey bees at the WWR, and to discern any interesting patterns in virus prevalence and titers in that population over time. In addition, we aimed to compare viral loads between wild colonies at the WWR and those from the nearest managed apiary. We hypothesized that, given the difference in management status, wild colonies at the WWR would have lower viral titers than managed colonies.

## Materials and Methods

### Study Site

This study was conducted at the WWR located in San Patricio County, TX (28°07ʹ 18″, −97°26ʹ34″). The WWR is positioned in a transitional zone between 3 different ecoregions: the South Texas plains, the Gulf prairies, and the marshes (Blankenship 2000). The vegetative cover is comprised of live oaks, mesquites, chaparral brushland, and open grassland (Blankenship 2000). The climate is generally humid, with hot summers and cool winters (Blankenship 2000, Baum 2003). Interestingly, Texas experienced a historical “polar vortex” in the winter of 2021 (before our last sampling event) that was accompanied by unusually intense cold, snow, and wind storms (Evett et al. 2021), arguably making it the worst cold spell in the region in over 100 years.

### Sample Collection from Tree Cavities

**Fig. 1.**
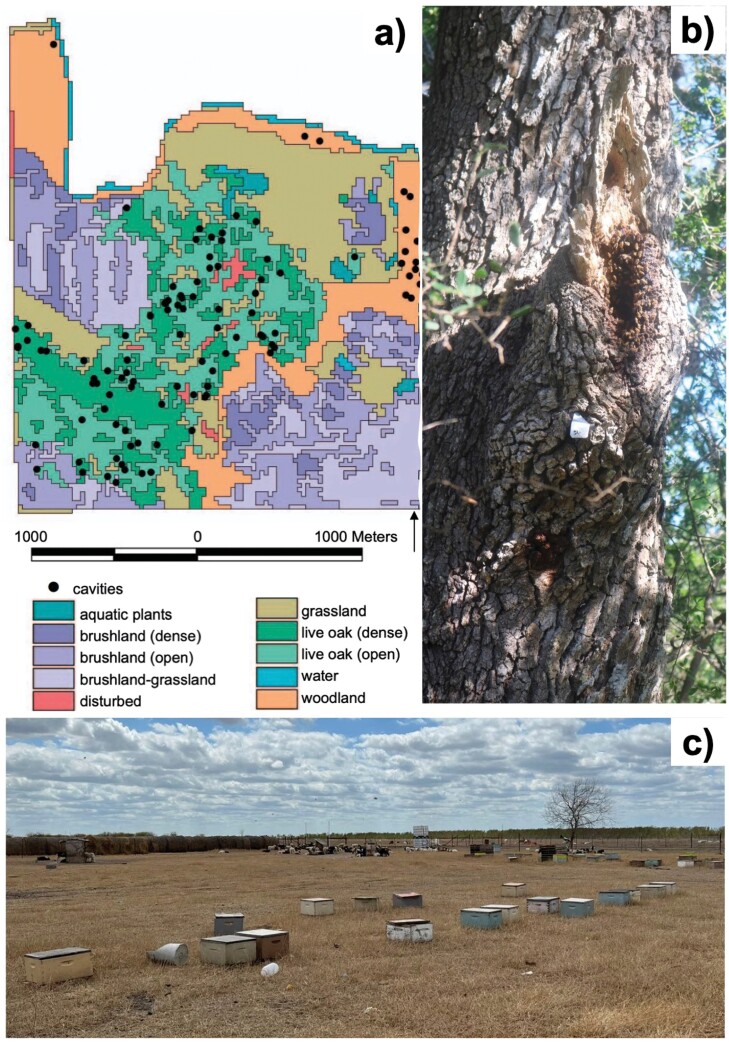
Images of a) the geographic layout of the Welder Wildlife Refuge (WWR) with the different types of vegetative cover (colored areas) and the tree cavities (black dots) that have been found harboring wild honey bee colonies (taken from Baum et al. 2005); b) a typical entrance of a tree cavity housing a wild honey bee colony at the WWR; and c) experimental colonies sampled at the nearest managed apiary from the WWR.

A thorough survey of wild honey bee colonies at the WWR conducted in 2013 and again in 2016 led to the discovery of 112 tree cavities in a 5.14 km^2^ area (Fig. 1a and b). All trees with suitable nesting cavities (most of which were live oaks) were marked with a metal tag and their global positioning system (GPS) coordinates were logged. We conducted a similar survey in 2021 and located some of the previously found trees by matching their tags to their GPS coordinates. We also marked and logged any new cavities harboring active colonies within the study area. We conducted all sample collections during the month of August to limit seasonal variation of virus prevalence or titers between the colonies that had been previously sampled in 2013 and 2016, and those that were newly sampled in 2021. We collected as many workers as possible from all active colonies by knocking on the entrance with a stick and catching any bees flying out of the hive. On average, about 15 bees were collected from the entrance of the most accessible colonies. However, because some of the cavities were not very accessible and were several meters above ground, we had difficulty collecting individuals from them. All collected bees were immediately placed on dry ice and transported back to the laboratory to be stored at −80°C for subsequent DNA and RNA extraction.

### Sample Collection from Managed Colonies

We partnered with a beekeeper located 30 miles away from WWR who maintains approximately 100 colonies (Fig. 1c). The beekeeper’s main commercial activities are queen rearing and honey production, and the colonies are rarely moved away from the site. The proprietor implements typical beekeeping procedures such as splitting, treating colonies against *Varroa* mites using approved chemical products, and requeening when colonies undergo queen supersedure or are failing. We collected approximately 20 workers from the entrance of 20 randomly chosen colonies, placed them on dry ice, and transported them back to the laboratory to be stored at −80°C for subsequent genetic analysis. In addition, there were 2 managed colonies maintained at the WWR that were sampled in 2021 for virus detection and quantification. Those colonies had been started a few years before by installing into a box a typical “package” of purchased bees that contained 2–3 Lb. of workers, along with a queen of European genetic ancestry. The owner of the colonies then took a “hands-off” approach to colony management, and by the time we sampled them in 2021, they had not been manipulated in over a year.

### DNA Extraction and Mitochondrial DNA Analysis

To determine the maternal genetic lineage of sampled colonies, total DNA was extracted from the thorax of one randomly selected worker per colony sampled at the WWR in 2013 and 2016 using a QIAamp DNA Mini Kit (Qiagen Inc., Valencia, CA), following the manufacturer’s specifications (see Rangel et al. 2016a for details on how DNA was extracted from those colonies). In addition, in 2021 DNA was extracted from the legs and wings of two randomly selected workers per colony following the Chelex method (Evans et al. 2013). Total DNA was extracted from workers belonging to the wild colonies sampled at the WWR, those “managed” colonies that were maintained at the WWR, and those managed colonies at the nearest managed apiary. We used 2 different DNA extraction methods because the DNA extracted in 2013 and 2016 (but not the DNA extracted in 2021) was done previously for a separate study (Rangel et al. 2016a). All extracted DNA was stored at −20°C until further analysis. We amplified the intergenic region of the *CO-I* and *CO-II* gene in the mitochondrial genome via polymerase chain reaction (PCR) by using the primers E2, 5’ GGACTAATATCTATACCACG 3’, and H2, 5’ CCTAAAGATGGAACTGTTC 3’, as done previously (Garnery et al. 1992).

We performed single PCR reactions in 50-μL volumes using the OneTaq kit from New England Biolabs (Ipswich, MA). Reactions contained the following reagents: 1X Standard Buffer, 200 μM of dNTPs, 0.2 μM of both forward and reverse primers, 1.25 units of OneTaq DNA polymerase, and 5 μL of 10-fold diluted DNA. The PCR thermocycler conditions were 94°C for 30 s followed by 29 cycles of 94°C for 15 s, 48°C for 20 s, 68°C for 45 s, and a final extension period of 68°C for 10 min. Following PCR, the products were separated on a 1% agarose gel stained with ethidium bromide and visualized under UV light. Once the presence of PCR products was confirmed, they were cleaned using Mag-Bind Total Pure NGS (Omega Bio-Tek, Norcross, GA). Cleaned PCR products were then sent off for sequencing at Eurofins Genomics (Louisville, KY). The forward and reverse reads were aligned to each other and were then used for carrying out nucleotide BLAST algorithms in the National Center for Biotechnology Information (NCBI) database for *A. mellifera* mitochondrial haplotype (mitotype) identification.

### RNA Extraction and cDNA Synthesis

To determine the presence and prevalence of selected honey bee-associated RNA viruses, RNA was extracted from a pool of 10 bees per colony. However, this was done only for wild colonies sampled at the WWR in 2013, 2016, and 2021 for which we had collected at least 10 bees, as we were unable to sample enough workers from some of the colonies. RNA was likewise extracted from a pool of 10 bees per colony for 20 randomly selected colonies at the managed apiary. For each of those colonies, RNA was extracted and homogenized using a mortar and pestle following the Ribozol (VWR Life Sciences, Radnor, PA) protocol. The concentration and purity of the RNA were measured on a Nanodrop2000 Spectrophotometer (Thermo Scientific, Wilmington, DE). Next, 100 ng of RNA was used to generate cDNA. Genomic DNA was then removed, and cDNA was synthesized for each sample using the iScript clear gDNA synthesis kit (Bio-Rad Laboratories, Hercules, CA). The resulting cDNA was then diluted 10-fold and stored at −20°C until it was used as a template for quantitative PCR (qPCR) amplification to identify each virus and quantify its relative abundance.

### Quantification of Viruses

Each RNA sample was first screened for the presence of 8 common honey bee-associated viruses: ABPV, BQCV, Chronic bee paralysis virus (CBPV), DWV-A, IAPV, KBV, Lake Sinai virus (LSV), and Sacbrood virus (SBV), using the primers outlined in Table 1. We performed 25-μL reactions using a GoTaq kit from Promega (Madison, WI). Each reaction contained 1X GoTaq master mix, 0.2 μM of both forward and reverse primers, and 1 μL of diluted cDNA. Each PCR product was separated using gel electrophoresis on a 3.5% agarose gel stained with ethidium bromide and visualized under UV light. Each colony was then scored as testing positive or negative for each of the 8 viruses.

**Table 1.** List of forward and reverse primers (and their respective source of reference) used for the detection and quantification via quantitative polymerase chain reactions for honey bee-associated viruses

Virus	Forward primer	Reverse primer	Reference
ABPV	ACCGACAAAGGGTATGATGC	CTTGAGTTTGCGGTGTTCCT	Boncristiani et al. 2012
BQCV	TTTAGAGCGAATTCGGAAACA	GGCGTACCGATAAAGATGGA	Boncristiani et al. 2012
CBPV	CAAAATCAACGAGCCAATCA	AGTGTGAGGATCACCGGAAC	Boncristiani et al. 2012
DWV	GAGATTGAAGCGCATGAACA	TGAATTCAGTGTCGCCCATA	Boncristiani et al. 2012
IAPV	GCGGAGAATATAAGGCTCAG	CTTGCAAGATAAGAAAGGGGG	Boncristiani et al. 2012
KBV	TGAACGTCGACCTATTGAAAAA	TCGATTTTCCATCAAATGAGC	Boncristiani et al. 2012
LSV	GTCATCCCAAGAGAACCACTYAC	CRCACYGACATGAAGAAATGAGGTC	Stamets et al. 2018
SBV	GGGTCGAGTGGTACTGGAAA	ACACAACACTCGTGGGTGAC	Boncristiani et al. 2012

To quantify virus titers, 2-step qPCR was performed in 96-well plates on all samples that tested positive for any given virus using a Bio-Rad CFX96 machine (Bio-Rad Corp, Hercules, CA). To do this, 1 μL of diluted cDNA was added to 5 μL of SYBR Green master mix (Applied Biosystems, Waltham, MA). Then, 10 μM of forward and reverse virus-specific primers were added. Each sample was performed in triplicate reactions, as well as having simultaneous water and no-reverse transcriptase reactions as negative controls. Amplification was performed using 39 cycles of 94°C for 2 min, 94°C for 30 s, 60°C for 30 s, and 72°C for 30 s, followed by a melting curve from 55°C to 95°C. The final RNA copy number of each virus per sample was calculated by extrapolation to a standard curve made by serial dilutions (1:10) of a viral fragment used as a reference, which was provided to us by the USDA’s Honey Bee Laboratory in Beltsville, MD.

### Statistical Analysis

For cases in which the same virus was detected in samples collected in all 3 years (2013, 2016, and 2021) from wild colonies sampled at the WWR, we conducted analysis of variance (ANOVA) tests to determine if there were differences in virus titers across years, followed by pair-wise Tukey honestly significant difference (HSD) tests. We also conducted Shapiro tests to determine if the quantification data for each identified virus were normally distributed. Because the data were not normally distributed, we performed Wilcoxon rank sum tests to determine if there were differences in virus titers between samples collected at the WWR and those collected from managed colonies in 2021. This comparison was only done for samples collected in 2021 because we did not collect samples from managed colonies in 2013 or 2016. Viral titers are reported as the triplicate average log_10_ viral copy number per sample (± the standard error of the mean (SEM)), as done previously (Tantillo et al. 2015, Posada-Florez et al. 2019). Virus infection is generally considered to be at low intensity when it ranges from 0 to 10^3^ copies, at medium intensity when it ranges from 10^3^ to 10^7^ copies, or at high intensity when it is above 10^7^ copies per sample (Amiri et al. 2015). All descriptive statistics are reported as the mean ± SEM. We set the level of statistical significance for all tests at α = 0.05.

## Results

### Sample Collection

Of the 112 tree cavities surveyed in 2013 and 2016, 28 harbored active colonies in 2013 and 36 harbored active colonies in 2016. Because some of the workers collected from those colonies were used in previous studies (Rangel et al. 2016a, b), the total number of colonies that we were able to use for virus detection was only 6 in 2013 and 24 in 2016. In 2021, only 16 of the cavities surveyed in previous years harbored active colonies, but we also found 4 additional cavities that had not been sampled before (for a final sample size of *n* = 20). We tagged those trees and recorded their GPS coordinates. Those were the only tree cavities that we were able to locate and sample from during our weeklong survey of the refuge.

### Detection, Prevalence, and Quantification of Viruses

Of the 8 viruses analyzed, CBPV, IAPV, and KBV were not detected in any of the tested colonies during any of the years sampled. Of the 6, 24, and 20 wild colonies at the WWR that were tested for viruses in 2013, 2016, and 2021, respectively, 4 (66.7%), 12 (50.0%), and 5 (25.5%) tested positive for at least one virus, respectively (see Supplementary Table S1 for the full list of the viruses that were detected in each colony in a given year). In 2013 and 2021, DWV and BQCV were the most prevalent viruses detected. In 2016, BQCV was the most prevalent virus detected. There was no difference over time in the prevalence of any of the viruses at the WWR (χ^2^ = 6, *P*-value = 0.199). Some colonies were infected with multiple viruses, which is not uncommon (Chen et al. 2004, 2005). For example, at least 2 viruses were found in 2 wild colonies sampled in 2013, 5 wild colonies sampled in 2016, and 3 wild colonies sampled in 2021 (see Supplementary Table S2 for the full list). In addition, 7 of the 20 managed colonies (35.0%) sampled from the nearest managed apiary tested positive for at least one virus (Supplementary Table S2). We found no difference in the prevalence of viruses between colonies from the nearby managed apiary and those at the WWR (χ^2^ = 0, *P *= 1.00).

We also found that viral titers in the wild honey bee population varied from year to year, displaying different infection titers depending on the virus and the year. For example, the triplicate sample average (±SEM) titer of DWV in wild bees at the WWR was 8.59 × 10^4^ ± 4.81 × 10^4^ log_10_ copy numbers in 2013 (*n* = 3 colonies that tested positive), 2.86 × 10^4^ ± 3.83 × 10^4^ log_10_ copy numbers in 2016 (*n* = 4), and 1.33 × 10^4^ ± 1.12 × 10^4^ log_10_ copy numbers in 2021 (*n* = 4), all of which are considered to be medium titers (see Figure 2a in Amiri et al. 2015). The average titers of DWV were significantly different across years (ANOVA, *F*_2,10_ = 8.38, *P < *0.001). Furthermore, DWV titers in 2013 were statistically higher than those in 2016 (Tukey HSD test, *P *< 0.001) and 2021 (Tukey HSD test, *P *< 0.001). There was no difference in average DWV titers between colonies sampled in 2016 and 2021 (Tukey HSD test, *P *= 0.691).

**Fig. 2.**
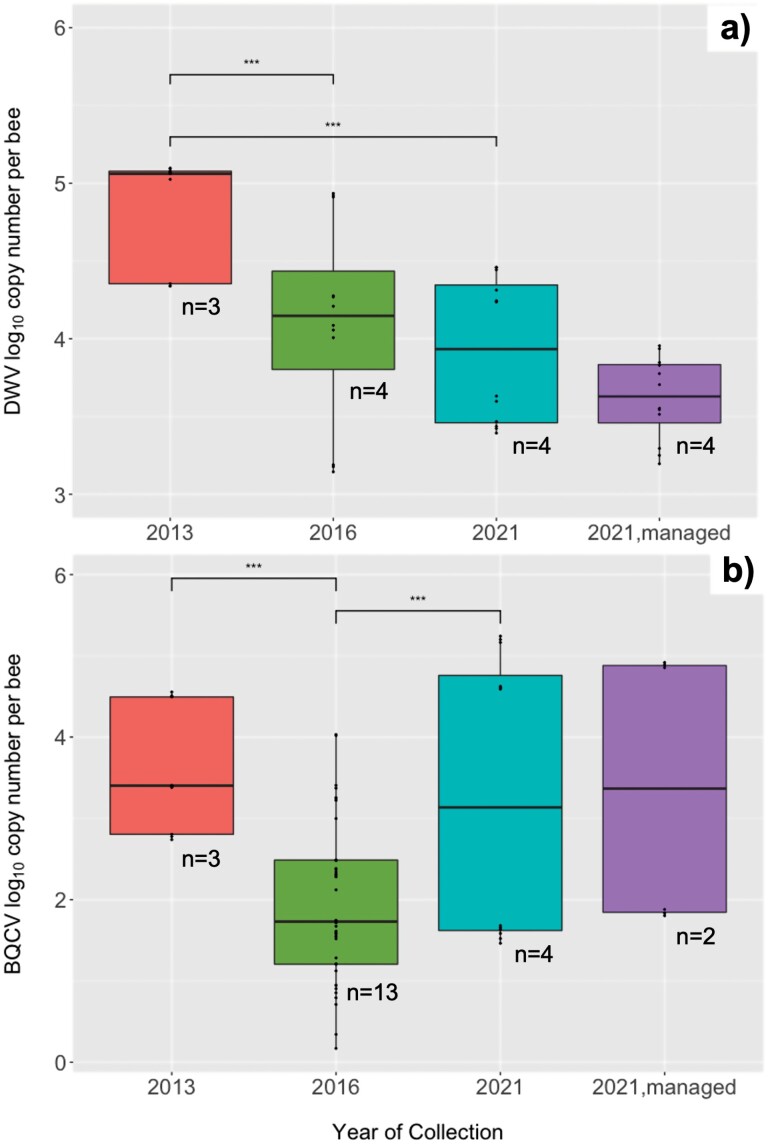
Boxplots showing the log_10_ of the viral copy number per bee (± the spread of all the data points, with each point shown by a black dot) for a) DWV and b) BQCV found in honey bee workers collected from colonies at the Welder Wildlife Refuge (WWR) in 2013 (salmon), 2016 (green), and 2021 (teel), as well as those collected from managed colonies sampled at the nearest apiary from the WWR in 2021 (purple). The number of colonies that tested positive for each virus in each year (*n*) is given below each box. There were 3 technical replicates analyzed per year per sample. Horizontal lines in each box represent the median. The “***” symbols above brackets denote a *P-*value < 0.001 when comparing virus titers between 2 specific years.

The triplicate sample average (±SEM) titer of BQCV infection was 1.21 × 10^4^ ± 1.58 × 10^4^ log_10_ copy numbers in 2013 (*n* = 3 colonies), 1.93 × 10^3^ ± 2.87 × 10^3^ log_10_ copy numbers in 2016 (*n* = 13), and 5.02 × 10^4^ ± 6.88 × 10^4^ log_10_ copy numbers in 2021 (*n* = 4), all of which are considered of low to medium titers (Amiri et al. 2015; Fig. 2b). The average titers of BQCV were significantly different across years (ANOVA, *F*_2,19_ = 9.32, *P < *0.001). BQCV titers of samples collected in 2013 were statistically higher than those collected in 2016 (Tukey HSD test, *P *< 0.001); they were also higher in samples from 2021 compared to those collected in 2016 (Tukey HSD test, *P *< 0.001). Furthermore, there was no difference in average BQCV titers between samples collected in 2013 and those from 2021 (Tukey HSD test, *P *= 0.819). SBV was only detected at low titers in WWR samples from 2016, with a triplicate sample average (±SEM) titer of 1.25 × 10^3^ ± 1.18 × 10^3^ copy numbers per sample (*n* = 2). LSV was detected from samples in 2013 with a triplicate sample average (±SEM) titer of 8.31 × 10^4^ ± 5.76 × 10^3^ copy numbers in 2013 (*n* = 1) and a triplicate sample (±SEM) titer of 5.41 × 10^4^ ± 2.40 × 10^3^ copy numbers per in 2021 (*n* = 1), both of which are considered medium intensity (see Figure 2a in Amiri et al. 2015).

We found no difference in DWV titers in managed colonies when including versus excluding the 2 managed colonies that were sampled at the WWR (Fig. 2; Wilcoxon rank sum test, *W* = 48, *P* = 0.698). Therefore, we pooled the DWV data from both managed locations in our analysis to increase the sample size slightly. DWV was found at relatively medium intensity in samples collected from the pooled samples from managed colonies, with a triplicate sample average (±SEM) titer of 4.84 × 10^3^ ± 2.62 × 10^3^ copy numbers (*n* = 4 colonies total). We found no differences in DWV titers between the wild and managed colonies sampled in 2021 (Wilcoxon rank sum test, *W* = 48, *P*-value* *= 0.175). Furthermore, BQCV was found at relatively medium intensity only in samples from the managed apiary at the WWR, with a triplicate sample average (±SEM) titer of 3.87 × 10^4^ ± 4.24 × 10^4^ copy numbers (*n* = 2), while no BQCV was detected in any of the colonies tested for viruses from the nearby managed apiary (*n* = 19 colonies for which there were enough bees to perform the test). There were no differences in BQCV titers between wild and managed colonies sampled at the WWR in 2021 (Wilcoxon rank sum test, *W* = 45, *P* = 0.426). We found low titers of ABPV in the nearby managed apiary samples, with a triplicate sample average (±SEM) titer of 2.52 × 10^1^ ± 2.50 × 10^1^ copy numbers (*n* = 3). ABPV was not detected in any of the samples collected at the WWR in 2021. Finally, LSV was found at medium intensity at the off-site managed apiary, with a triplicate sample average (±SEM) titer of 1.97 × 10^6^ ± 6.56 × 10^5^ copy numbers (*n* = 1).

### Mitochondrial Haplotype Analysis

Mitochondrial haplotypes for all colonies sampled at the WWR in 2013 were previously published in Rangel et al. (2016a). Of the 28 colonies sampled that year, 5 (83%) belonged to the A lineage (corresponding to the *Apis mellifera scutellate* subspecies) and one (27%) belonged to the C lineage. However, we were able to perform virus detection and quantification from only 6 of the 28 colonies sampled in 2013 (21.4%) because we did not have enough bees from each of the other colonies for analysis. Likewise, we only report haplotypes for 24 of the 36 colonies sampled in 2016 (66.7%), which correspond to those for which we had enough bees to conduct viral identification and quantification. Of the 24 colonies analyzed from 2016, 22 (92%) belonged to the A lineage, one colony (4%) belonged to the O lineage, and one colony (4%) was scored as there being no mitotype match for it in the NCBI database. For bees collected at the WWR in 2021, we only reported the haplotypes of 19 of the 20 colonies sampled because those were the colonies for which we had enough bees to perform viral identification and quantification. Of those 19 colonies analyzed for viruses, 17 (90%) belonged to the A lineage and 2 (10%) belonged to the O lineage (Supplementary Fig. S1). The haplotypes of wild colonies sampled from the WWR in 2016 and 2021 are listed in Table 2. Finally, of the 20 colonies from the nearby managed apiary that were analyzed, 17 (85%) belonged to the C lineage and 3 (15%) belonged to the A lineage (Supplementary Fig. S1). Both “managed” colonies (100%) that are maintained on the WWR property belonged to the A lineage. The haplotypes for colonies sampled at the managed apiary, as well as those of the 2 managed colonies at the WWR, are listed in Table 3.

**Table 2.** List of wild honey bee colonies sampled at the Welder Wildlife Refuge in 2016 and 2021, along with their corresponding mitochondrial haplotype (mitotype) identification

Colony ID	Mitotype in 2016	Mitotype in 2021
60	A26a	29a
76.2		A4p
78	A1	
83	A1e	
95		A4p
301		A1
349	A1e	
356		A1e
357	A26a	A4p
359	A1e	A1e
366	A26	
370	A1e	
379		A1e
381		A4p
383	A26a	O5
384	A1e	A4p
385	A16’	
393	A1e	
394		A4p
396	A1e	
403	O	
404	A16’	29a
406	A16’	
407	A1e	
418	A1e	
425	A1e	A4p
427	A1e	A1e
428	A1e	
435	A1e	A1e
436	No match	
501	A1e	
517		A1e
703		O5
704		A26
721		A1e

**Table 3.** List of managed honey bee colonies that were screened in 2021 for select honey bee-associated viruses, along with their corresponding mitochondrial haplotype (mitotype) identification. Colonies identifications (IDs) starting with D denote those that were sampled at the nearest managed apiary from the WWR, while colony IDs that start with T denote the two colonies that were managed on-site at the WWR

Colony ID	Mitotype
D1	A1e
D2	A1e
D3	A1e
D5	C2
D6	C2
D7	C2
D8	C2
D9	C2
D11	C2
D12	C2
D13	C2
D14	C2
D15	C2
D16	C2
D17	C2
D18	C2
D19	C2
D20	C2
D21	C2
D22	C2
T1	A1e
T2	A1e

## Discussion

This study comprised 3 main goals. First, we wanted to do a preliminary survey of the prevalence and titers of selected RNA viruses in wild honey bee colonies located at the WWR. Second, we wanted to identify any possible virus infection patterns in colonies sampled from this population across multiple years. And third, we wanted to compare the presence and titers of viruses between wild colonies at the WWR and those from the nearest managed apiary. Our results showed that DWV and BQCV, 2 of the biggest viral threats to managed honey bees (Ullah et al. 2021), were indeed detected in wild colonies at the WWR. However, infection from both viruses is only considered to be of low to medium intensity (Amiri et al. 2015). Overall, virus titers of the 2 most prevalent viruses, DWV and BQCV, were significantly different in wild colonies across years (Fig. 2a and b, respectively). There were no differences in the prevalence of viruses over time at the WWR or between wild colonies and those from the nearby managed apiary. We saw a similar pattern of viral infection between DWV and BQCV, with titers for both viruses being highest in 2013 and then dropping in 2016. However, it should be noted that the number of colonies sampled at the WWR in 2013 that we could use for virus identification was low (*n* = 6), given the difficulty in collecting bees from remote hive entrances in many instances, and the fact that many of the collected bees had been used in a previous study (Rangel et al. 2016). Therefore, it would be interesting to know if having a larger sample size could have indicated even stronger statistical differences in DWV and BQCV titers over the years. Furthermore, while the titer of DWV infection in wild colonies sampled in 2016 was similar to those sampled in 2021, the titer of BQCV infection was significantly higher in 2021 than in 2016. This result is not surprising, given that viral prevalence and titers are known to fluctuate in managed colonies on a seasonal basis (Traynor et al. 2020), so this pattern could be also present in wild populations. Regardless of these shortcomings, our study provides some of the first information about how viral titers fluctuate in a wild population of honey bees over time.

Furthermore, we found no significant differences in the titers of DWV or BQCV between wild colonies at the WWR and managed colonies from the nearest off-site managed apiary. Interestingly, even though some of the wild bees tested positive for DWV and BQCV that year, the continued presence of active colonies in some of the tree cavities that bees had been sampled from in 2013 and 2016 (e.g., cavity # 383) is an indication that some of these colonies have survived without human intervention year after year, despite them having low to medium intensity of some viruses. However, while other studies have shown that wild colonies are expected to survive at a higher rate than managed colonies (Loftus et al. 2016, Seeley 2017), we were unable to sample each tree cavity more than once per year due to the remote location of the WWR. Therefore, we cannot definitively say that an active colony inhabiting a given tree cavity in 2013 was present and sampled from the same cavity in 2016 or 2021. In the future, studies of the turnover rate of colonies in wild settings should assess each tree cavity multiple times per year and perform genetic analyses to determine if the same active colony is present in the same cavity over time.

Overall, we know little about how virus infections fluctuate over time in free-living colonies compared to managed colonies. Comparable to our current results, a recent study in Southern California found that feral and managed colonies had similar titers of viral pathogens (Geffre et al. 2021). In particular, the authors found that wild and managed colonies were infected at medium intensity with DWV and BQCV, while ABPV infection fluctuated between low and medium intensity. Conversely, 2 recent studies (1 in the United Kingdom and 1 in Pennsylvania) found that DWV titers were higher in feral colonies compared to managed colonies (Thompson et al. 2014, Hinshaw et al. 2021). While the titers of DWV that we found in wild colonies at the WWR were slightly higher than those in the managed population, the difference was not statistically significant. Notably, each of the 3 studies mentioned above (Thompson et al. 2014, Geffre et al. 2021, Hinshaw et al. 2021) defined “feral” colonies as those that would have survived in unmanaged conditions for at least 1 full year. This means that some of the colonies that those studies considered to be feral could have come from escaped swarms that had recently escaped from nearby apiaries and thus, they would have only lived in unmanaged conditions for a year or less. On average, a well-established honey bee colony will swarm with its mother queen once a year in the spring, during which time there is usually a break in the brood-rearing cycle until the new queen begins laying eggs (Winston 1987). This interrupts the life cycle of the *Varroa* mite, therefore decreasing the number of mites in the colony, and potentially also reducing the transmission of viruses (Seeley 2017). This “natural” method to reduce pathogen loads is generally inhibited in managed beekeeping operations because apiary managers instead prefer to use pesticides for *Varroa* control. In addition, commercially managed hives have been shown to impact viral dynamics (often causing higher virus prevalence) than feral colonies (Owen 2017, Bartlett et al. 2021). Therefore, considering a colony to be “feral” only if they have survived unmanaged for a year is, in our opinion, likely not long enough for natural selection to effectively cull out colonies not well suited to withstand the pressures of living in the wild without any husbandry.

Here we also document the mitotypes of colonies sampled at the WWR in 2016 and 2021 (the mitotypes from colonies sampled in 2013 had previously been reported in Rangel et al. 2016). The vast majority of the colonies sampled (90–92%) belonged to the A (African) mitotype, with the A1e version being the most common (Table 2). Only 1 or 2 colonies (4–10%) were of the O (Middle Eastern) lineage, a mitotype that is not very common in commercial beekeeping operations (Magnus et al. 2011). A recent study of a feral population in Southern California also reported a few colonies (4 out of 15) belonging to the O lineage (Zárate et al. 2022). Combined, these interesting findings suggest that wild populations of honey bees in the southern United States harbor an untapped resource of genetic diversity that should be further explored in the future. It is also interesting to note that, of the 4 colonies from the nearby managed apiary that belonged to the A linage in our study, none tested positive for any viruses.

While the 2 onsite “managed” colonies at the WWR were also of the A lineage and tested positive for DWV and BQCV, those colonies have not been actively taken care of by beekeepers in years, so the bees that live in those boxes could be swarms that moved into those boxes without anybody noticing. Incidentally, our anecdotal data show that, over time, colonies that are found in the same tree cavity in consecutive years do not always have the same mitotype as those sampled in prior years. This pattern was previously documented in the 1980s and early 2000s when the WWR population was primarily of European maternal ancestry and then, after the introduction of the *Varroa* mite, colonies of the European linage were displaced in favor of those of primarily African maternal ancestry (Pinto et al. 2005). Since the population of unmanaged colonies at the WWR has become primarily of Africanized descent, it seems that the A lineage is the best-suited mitotype for the ecological conditions in this part of the southern United States. Zárate et al. (2022) similarly found that most of the feral colonies (60%) they sampled in Southern California were of the A maternal lineage.

Colonies belonging to the A maternal lineage continue to dominate the landscape at the WWR despite extreme environmental phenomena such as the historically devastating regional drought in 2011 (Combs 2012), and the cold temperatures during the “polar vortex” event in the winter of 2021. Indeed, previous work has shown that locally adapted honey bees are better suited to environmental fluctuations than non-local colonies (Costa et al. 2012, Hatjina et al. 2014). The wild colonies at the WWR are a unique beacon of genetic diversity because they have truly lived in unmanaged conditions for more than 30 years and are located over 30 miles away from the closest managed beekeeping operation (Pinto et al. 2003, 2004, 2005, Rangel et al. 2016a, 2016b). This makes it unlikely that any of the WWR colonies are swarms that recently escaped from nearby apiaries (except for the 2 on-site “managed” colonies, which are not actively taken care of by beekeepers). Furthermore, wild colonies at the WWR have never been treated against *Varroa* or any other parasites or pathogens. This lack of management has likely allowed for natural selection to act upon this population, which could explain why some colonies were infected with viruses at medium intensity and still survived to this day. This is a main response of pathogen-host interactions which has allowed these honey bee colonies to survive under stress.

Managed colonies are generally kept in controlled conditions (a vastly different lifestyle than that of wild colonies), which can mask or reverse the evolutionary pressures they are faced with due to human intervention. For example, most managed colonies are treated on an annual basis against high *Varroa* infestations, mostly through the use of pesticides (Jack and Ellis 2021). These constant treatment regimens disrupt natural selection for mite- and/or virus-tolerant colonies (Neumann and Blacquière, 2017). Managed colonies with high *Varroa* numbers typically also exhibit high titers of viruses (Carreck et al. 2010, Toufailia et al. 2014, Emsen et al. 2015). Managed colonies are also typically located at high densities in small areas, which can increase transmission of *Varroa* and potentially other pathogens (Seeley and Smith 2015). In contrast, our results suggest that wild colonies at the WWR might have low *Varroa* infestations, given that they exhibit low to medium viral titers. In addition, the frequent swarming and small colony size of wild Africanized colonies could play a role in keeping mite numbers and viral titers low (Winston et al. 1983). Furthermore, Hinshaw et al. (2021) found that feral colonies have higher expression of immune genes than their managed counterparts. Potentially, wild colonies at the WWR also have higher expression of immune genes, which could make them more tolerant to pests and pathogens. This concept should be tested further in future studies.

While our study provides some of the first information about the titers of viral pathogens in a truly wild honey bee population in the southern United States, there are some caveats in our experimental design that need to be considered. First, we only collected foragers from the hives’ entrances because we were not allowed to destructively sample bees from inside the hives. Also, given that some of these tree cavities were either located several meters above ground, were very aggressive when disturbed, or were in a difficult location to collect samples, we were unable to collect many bees per colony. Therefore, for virus quantification, we decided to pool 10 bees per colony to increase the number of total colonies screened, instead of pooling a collective 20–30 individuals, which is recommended by Pirk et al. (2013). However, the probability of detecting pathogens only increases by 4% when adding more individuals to a pool of 10 (Pirk et al. 2013). Nonetheless, if a virus was present at low titers in one of our sampled colonies, we could have gotten a falsely negative result in our analysis. If this was the case, potentially more of the wild colonies we sampled could have been infected with viruses at low titers, which our pooling of only 10 individuals per sample was unable to detect. Having said that, we chose not to pool 20 or more individuals per colony for virus quantification because this would have cut our sample size by half, going from 20 screened colonies to only 8.

Second, because we did not collect enough bees from a hive’s entrance, we were unable to perform suitable *Varroa* mite counts, which require sampling at least 300 adult bees from the middle of the brood nest area, where the highest number of mites on adult bees can be found (Dietemann et al. 2013). Also, since only foragers were collected, and we were unable to sample from the brood nest area, we only obtained one snapshot of a colony’s adult population. However, a recent study compared the viral titers of individual foragers to those of the entire colony and found higher titers of BQCV and CBPV in foragers compared to the rest of the colony (Penn et al. 2022).

Finally, we were unable to confirm if the same colony was present in a given tree cavity for multiple years, as we could not go inside the hive to monitor the turnover rate of new queens. Even though some colonies in the same tree cavity did maintain the same mitotype from one year to the next, we cannot definitively say that these were the same colonies surviving over multiple years. For instance, we were unable to sample inside the colony to determine if the same queen was present across multiple years, and were also unable to determine if a colony swarmed and re-occupied an available cavity, which would allow a sampled colony to have the same mitotype as that in a previous year, but it would not necessarily be from the same queen. We did return to the WWR in the spring of 2022 to sample bees for another study and found that only 2 of the colonies sampled in 2021 were either too weak to sample, or had died. So, overall, we only saw a 10% “loss” of colonies in 2022 compared to the number of active colonies that we found in 2021. While we are unsure if those colonies were truly lost or if they relocated someplace else, this low rate of loss within a 6-month period suggests resilience in some of these wild colonies. Future work should investigate colony survivorship and tree cavity turnover at the WWR year-round and over time.

Despite the aforementioned limitations, it is evident that wild colonies at the WWR only suffered from low to medium infection titers of the tested RNA viruses, potentially indicating that these colonies have higher tolerance to pathogen pressure compared to managed colonies. This is especially important when one considers that the average annual loss of managed colonies in the United States has been upwards of 30% year after year (Kulhanek et al. 2017, Bruckner et al. 2023), even though most colonies are heavily treated against parasites, pathogens, and pests. Given the importance of honey bees for human welfare, it is now even more important to study the population genetics and disease ecology of wild honey bee populations to understand how they are able to combat pathogens and thrive without human intervention.

## Supplementary Material

iead117_suppl_Supplementary_Tables_S1

iead117_suppl_Supplementary_Tables_S2

iead117_suppl_Supplementary_Figures_S1

## Data Availability

The original contributions presented in this study are included in the article and/or the Supplementary material. Further inquiries can be directed to the corresponding author. The datasets generated and analyzed for this study can be found at the Texas Data Repository by visiting https://doi.org/10.18738/T8/IAMHI4.
