# Supplementary material for: A preliminary survey reveals that common viruses are found at low titers in a wild population of honey bees (Apis mellifera)
Source: J Insect Sci. 2023 Dec 14;23(6):26. doi: 10.1093/jisesa/iead117 (PMC10721442; doi:10.1093/jisesa/iead117)
Supplement: iead117_suppl_Supplementary_Tables_S1 [file iead117_suppl_supplementary_tables_s1.docx]

| **Supplemental Table S1.** List of wild honey bee colonies (with their colony IDs) sampled at the Welder Wildlife Refuge in 2013, 2016, and 2021, as well as the viruses that were detected in each colony in a given year. BQCV = black queen cell virus, DWV = deformed wing virus, LSV = lake sinai virus, and n.d. = none detected. Blank cells refer to cases in which a given tree cavity was not occupied by an active colony in a given year.   \|  \| \| --- \| |  |  |  |
| --- | --- | --- | --- | --- |
|  |  |  |  |
|  |  |  |  |
|  |  |  |  |
| **Colony ID** | **Viruses detected in 2013** | **Viruses detected in 2016** | **Viruses detected in 2021** |
| 60 |  | n.d. | n.d. |
| 76.2 |  |  | DWV, BQCV, & LSV |
| 77 | BQCV |  |  |
| 78 |  | DWV & BQCV |  |
| 83 |  | DWV & BQCV |  |
| 95 |  |  | n.d. |
| 301 |  |  | n.d. |
| 349 |  | BQCV |  |
| 356 |  |  | n.d. |
| 357 |  | BQCV | n.d. |
| 359 |  | n.d. |  |
| 366 |  | n.d. |  |
| 370 |  | BQCV |  |
| 379 |  |  | n.d. |
| 381 |  |  | n.d. |
| 383 | DWV | BQCV | DWV & BQCV |
| 384 | DWV & BQCV | BQCV | n.d. |
| 385 |  | n.d. |  |
| 393 |  | n.d. |  |
| 394 | DWV, BQCV, & LSV |  | n.d. |
| 396 |  | BQCV & SBV |  |
| 396#1 | n.d. |  |  |
| 403 | n.d. | n.d. |  |
| 404 |  | n.d. | DWV & BQCV |
| 406 |  | BQCV |  |
| 407 |  | DWV, BQCV & SBV |  |
| 418 |  | DWV & BQCV |  |
| 425 |  | BQCV | n.d. |
| 427 |  | n.d. | n.d. |
| 428 |  | n.d. |  |
| 435 |  | n.d. | n.d. |
| 436 |  | n.d. |  |
| 501 |  | n.d. |  |
| 517 |  |  | DWV |
| 703 |  |  | BQCV |
| 704 |  |  | n.d. |
| 721 |  |  | n.d. |
