# Supplementary material for: A preliminary survey reveals that common viruses are found at low titers in a wild population of honey bees (Apis mellifera)
Source: J Insect Sci. 2023 Dec 14;23(6):26. doi: 10.1093/jisesa/iead117 (PMC10721442; doi:10.1093/jisesa/iead117)
Supplement: iead117_suppl_Supplementary_Tables_S2 [file iead117_suppl_supplementary_tables_s2.docx]

| **Supplemental Table S2.** List of managed honey bee colonies sampled in 2021 and the viruses that were detected in each colony. Colony identifications (IDs) starting with D denote those that were sampled at the nearest managed apiary from the WWR, while colonies with IDs that start with T denote the two managed colonies at the WWR. ABPV = acute bee paraylis virus, BQCV = black queen cell virus, DWV = deformed wing virus, LSV = Lake Sinai virus, and n.d. = none detected.   \|  \| \| --- \| |  |
| --- | --- | --- |
|  |  |
|  |  |
|  |  |
|  |  |
|  |  |
|  |  |
|  |  |
|  |  |
|  |  |
|  |  |
|  |  |
| **Colony ID** | **Viruses detected** |
| D1 | n.d. |
| D2 | ABPV |
| D3 | n.d. |
| D5 | n.d. |
| D6 | n.d. |
| D7 | n.d. |
| D8 | n.d. |
| D9 | DWV |
| D11 | n.d. |
| D12 | n.d. |
| D13 | n.d. |
| D14 | DWV & ABPV |
| D15 | n.d. |
| D16 | LSV |
| D17 | n.d. |
| D18 | n.d. |
| D19 | n.d. |
| D20 | ABPV & LSV |
| D21 | n.d. |
| D22 | n.d. |
| T1 | BQCV |
| T2 | DWV & BQCV |
