# Supplementary figures and images for: A preliminary survey reveals that common viruses are found at low titers in a wild population of honey bees (Apis mellifera)
Source: J Insect Sci. 2023 Dec 14;23(6):26. doi: 10.1093/jisesa/iead117 (PMC10721442; doi:10.1093/jisesa/iead117)

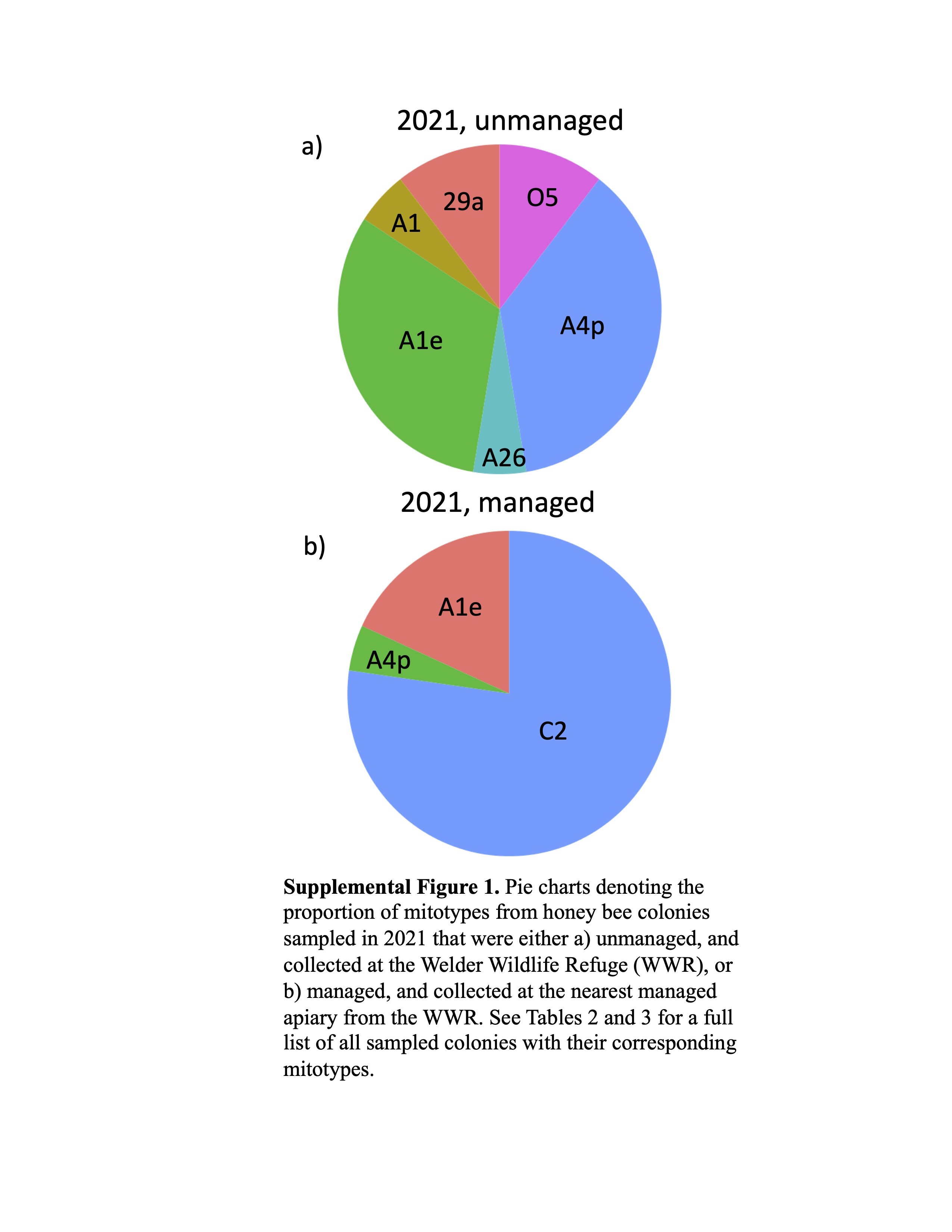

Supplement: iead117_suppl_Supplementary_Figures_S1 [file iead117_suppl_supplementary_figures_s1.jpeg]
